# Inhibition of proliferation and induction of apoptosis in soft tissue sarcoma cells by interferon-**α** and retinoids

**DOI:** 10.1038/sj.bjc.6690528

**Published:** 1999-07

**Authors:** T Brodowicz, C Wiltschke, D Kandioler-Eckersberger, T W Grunt, M Rudas, S M Schneider, M Hejna, A Budinsky, C C Zielinski

**Affiliations:** Clinical Division of Oncology, Chair of Medical Experimental Oncology, Department of Medicine I, Department of Surgery and Department of Clinical Pathology, University Hospital, and Ludwig Boltzmann Institute for Clinical Experimental Oncology, Vienna, Austria

**Keywords:** apoptosis, soft tissue sarcoma, interferon-α, retinoids, p53

## Abstract

Uncontrolled proliferation and a defect of apoptosis constitute crucial elements in the development and progression of tumours. Among many other biological response modifiers known to influence these mechanisms, the efficacy of retinoids and interferons in the treatment of various malignant entities is currently matter of discussion. In the present study, we have investigated the effects of 9-*cis*-retinoic acid (9cRA), 13-*cis*-retinoic acid (13cRA), all-*trans*-retinoic acid (tRA) and interferon-α on proliferation and apoptosis of human soft tissue sarcoma (STS) cell lines HTB-82 (rhabdomyosarcoma), HTB-91 (fibrosarcoma), HTB-92 (liposarcoma), HTB-93 (synovial sarcoma) and HTB-94 (chondrosarcoma) in relation to p53 genotype as well as p53 expression. HTB-91, HTB-92 and HTB-94 STS cells exhibited mutant p53, whereas wild-type p53 was found in HTB-93 STS cells, and a normal p53 status in HTB-82 STS cells, carrying a silent point mutation only. Interferon-α, irrespective of p53 status, inhibited the proliferation of all five cell lines dose- and time-dependently. Similarly, 9cRA, 13cRA and tRA decreased the proliferation of HTB-82 and HTB-93 STS cells, whereas the proliferation of p53-mutated HTB-91, HTB-92 and HTB-94 STS cells remained unchanged. Furthermore, only 9cRA and tRA were capable of inducing apoptosis in HTB-82 and HTB-93 STS cells, whereas HTB-91, HTB-92 and HTB-94 STS cells did not undergo apoptosis under the influence of 9cRA or tRA. Retinoic acid receptor (RAR)-α and RAR-β mRNA were not detectable by Northern blot analysis in the five STS cell lines, whereas mRNA for the universal retinoic acid receptor, RAR-γ, was expressed in all STS cell lines indicating that retinoid resistance was not associated with a lack of RAR expression. Apoptosis was not induced by interferon-α or 13cRA in any of the five STS cell lines tested. Our results indicate that within the panel of tested STS cell lines, inhibition of proliferation and induction of apoptosis result from different mechanisms which differ in their dependence upon the presence of intact p53. © 1999 Cancer Research Campaign


*British Journal of Cancer* (1999) **80**(9), 1350–1358
© 1999 Cancer Research Campaign
Article no. bjoc.1998.0528

### Inhibition of proliferation and induction of apoptosis in soft tissue sarcoma cells by interferon-α and retinoids

**T Brodowicz[1], C Wiltschke[1], D Kandioler-Eckersberger[3], TW Grunt[1], M Rudas[4], SM Schneider[1], M Hejna[1], A Budinsky[1] and CC Zielinski[1,2,5]**

[1]Clinical Division of Oncology and [2]Chair of Medical Experimental Oncology, Department of Medicine I, [3]Department of Surgery and [4]Department of Clinical Pathology, University Hospital, and [5]Ludwig Boltzmann Institute for Clinical Experimental Oncology, Vienna, Austria

**Summary** Uncontrolled proliferation and a defect of apoptosis constitute crucial elements in the development and progression of tumours. Among many other biological response modifiers known to influence these mechanisms, the efficacy of retinoids and interferons in the treatment of various malignant entities is currently matter of discussion. In the present study, we have investigated the effects of 9-*cis*-retinoic acid (9cRA), 13-*cis*-retinoic acid (13cRA), all-*trans*-retinoic acid (tRA) and interferon-α on proliferation and apoptosis of human soft tissue sarcoma (STS) cell lines HTB-82 (rhabdomyosarcoma), HTB-91 (fibrosarcoma), HTB-92 (liposarcoma), HTB-93 (synovial sarcoma) and HTB-94 (chondrosarcoma) in relation to p53 genotype as well as p53 expression. HTB-91, HTB-92 and HTB-94 STS cells exhibited mutant p53, whereas wild-type p53 was found in HTB-93 STS cells, and a normal p53 status in HTB-82 STS cells, carrying a silent point mutation only. Interferon-α, irrespective of p53 status, inhibited the proliferation of all five cell lines dose- and time-dependently. Similarly, 9cRA, 13cRA and tRA decreased the proliferation of HTB-82 and HTB-93 STS cells, whereas the proliferation of p53-mutated HTB-91, HTB-92 and HTB-94 STS cells remained unchanged. Furthermore, only 9cRA and tRA were capable of inducing apoptosis in HTB-82 and HTB-93 STS cells, whereas HTB-91, HTB-92 and HTB-94 STS cells did not undergo apoptosis under the influence of 9cRA or tRA. Retinoic acid receptor (RAR)-α and RAR-β mRNA were not detectable by Northern blot analysis in the five STS cell lines, whereas mRNA for the universal retinoic acid receptor, RAR-γ, was expressed in all STS cell lines indicating that retinoid resistance was not associated with a lack of RAR expression. Apoptosis was not induced by interferon-α or 13cRA in any of the five STS cell lines tested. Our results indicate that within the panel of tested STS cell lines, inhibition of proliferation and induction of apoptosis result from different mechanisms which differ in their dependence upon the presence of intact p53.

**Keywords:** apoptosis; soft tissue sarcoma; interferon-α; retinoids; p53

Soft tissue sarcomas (STS) represent a rare entity of malignant disorders of various histologies with mostly aggressive characteristics both locally and in the formation of distant metastases. In contrast to a variety of other malignancies and with exceptions reported in only a few trials and/or analyses (Gherlinzoni et al, 1986; Ravaud et al, 1990; Antman, 1997; Sarcoma Meta-Analysis Collaboration, 1997), adjuvant chemotherapy has not shown to significantly improve relapse-free and/or overall survival in patients with STS. Similarly, chemotherapeutic interventions in metastatic STS were generally also disappointing (Borden et al, 1990; Antman et al, 1993). Thus, cure of early disease obviously depends upon appropriate, well-designed interdisciplinary therapy of the primary tumour, including surgery, radiotherapy and – in some cases (Rosenberg et al, 1982; Brodowicz et al, 1997; Sarcoma Meta-Analysis Collaboration, 1997) – cytotoxic chemotherapy.

Based upon the above considerations of an obviously limited efficacy of chemotherapeutic drugs upon the control of proliferation and/or induction of apoptosis in STS, alternate approaches were sought. Although the underlying reasons have not been clarified, certain STS cell lines have been demonstrated to be inhibited in their proliferation by retinoids (Gabbert et al, 1988; Crouch and Helman, 1991) and/or interferon-α (IFN-α; Rosso et al, 1992) in vitro and in vivo. In order to follow and further expand these early experiments, and put them into perspective with recent insights into the regulation of proliferation inhibition and generation of apoptosis on the molecular level, we have investigated the induction of these phenomena by a series of retinoids (9-*cis*-retinoic acid (9cRA), 13-*cis*-retinoic acid (13cRA), all-*trans* retinoic acid (tRA)) and IFN-α in various human STS cell lines including HTB-82 (rhabdomyosarcoma), HTB-91 (fibrosarcoma), HTB-92 (liposarcoma), HTB-93 (synovial sarcoma) and HTB-94 (chondrosarcoma) in vitro and put them into context with p53 genotype as well as p53 protein expression. This was of particular interest, as growth arrest and induction of apoptosis resulting from appropiate chemo- and/or radiotherapeutic measures have been demonstrated to be dependent upon the intact function of the p53 gene in various models (Bergh et al, 1995; Elledge et al, 1995; Sarkis et al, 1995). Thus, patients with testicular tumours with regular p53 function have been shown to respond particularly well to chemotherapy (Lutzker and Levine, 1996), whereas patients with malignancies exhibiting high frequency of p53 mutations are known to present often with resistance to chemotherapeutic agents (Aas et al, 1996). In the present paper, we report that IFN-α induced inhibition of proliferation of all cell lines irrespective of

*Received 2 July 1998*
*Revised 4 November 1998*
*Accepted 8 December 1998*

*Correspondence to:* CC Zielinski, Department of Medicine I, Clinical Division of Oncology, University Hospital, Waehringer Guertel 18–20, A-1090 Vienna, Austria their p53 status, whereas retinoid-induced apoptosis was dose- and time-related, depended upon the presence of regular p53 and differed between retinoid preparations and, finally, STS cell lines.

### MATERIALS AND METHODS

### Cell cultures

Cell lines were obtained from American Type Culture Collection (ATCC). HTB-82 (A-204, human rhabdomyosarcoma) were cultured in McCoy's 5A medium supplemented with 10% heat-inactivated fetal calf serum (FCS; all from Gibco, Life Technologies Ltd, Paisley, UK), 50 U $ml^{-1}$ penicillin, 50 μg $ml^{-1}$ streptomycin and 2 mM L-glutamine (all from HyClone, Europe Ltd, Cramlington, UK). The human STS cell lines HTB-91 (fibrosarcoma, SW 684), HTB-92 (liposarcoma, SW 872), HTB-93 (synovial sarcoma, SW 982) and HTB-94 (chondrosarcoma, SW 1353) were cultured in Leibovitz's L-15 medium with L-glutamine (PAA Laboratories Gmbh, Linz, Austria) supplemented with 10% heat-inactivated FCS (Gibco), 50 U penicillin and 50 μg streptomycin (all from HyClone) per ml. HTB-91, HTB-92, HTB-93 and HTB-94 were grown as monolayers (standard conditions) in T75 flasks (Falcon, Becton Dickinson, NJ, USA) at 37°C in a humidified atmosphere with free gas exchange without carbon dioxide ($CO_2$) by seeding $5 \times 10^6$ cells in 25 ml of appropriate medium. HTB-82 cells were cultured in a humidified atmosphere containing 5%.

### Chemicals

9cRA, 13cRA and tRA (Biomol Research Laboratories, Inc., Plymouth Meeting, PA, USA) were dissolved in 1 ml dimethyl sulphoxide (Sigma, St Louis, MO, USA) at a concentration of $8.3 \times 10^{-5}$ M and then diluted with the appropriate culture medium (final concentrations: $10^{-7}$ M, $10^{-6}$ M and $10^{-5}$ M). Retinoid stock solution was kept at – 20°C. Experiments with retinoids were performed in subdued yellow light. Then, $5 \times 10^6$ IU IFN-α-2b (Schering-Plough NJ, USA) was dissolved in 1 ml of distilled water and subsequently diluted in appropriate culture medium (final concentrations: 25 U $ml^{-1}$, 50 U $ml^{-1}$, 100 U $ml^{-1}$ and 1000 U $ml^{-1}$).

**Table 1** p53 gene mutations characterized in five human soft tissue sarcoma cell lines

| Cell line | Base change | Exon/codon | Amino acid change |
|---|---|---|---|
| HTB-91 (fibrosarcoma) | ATC to AAC | 7/251 | *Ile–Asn* |
| HTB-92 (liposarcoma) | GTG to TTG | 6/203 | *Val–Leu* |
| HTB-94 (chondrosarcoma) | CGA to TGA | 6/213 | *Arg*–STOP |
| HTB-94 (chondrosarcoma) | TTC to TTT | 10/341 | *Phe–Phe*; silent |
| HTB-82 (rhabdomyosarcoma) | CGA to CGG | 6/213 | *Arg–Arg*; silent |
| HTB-93 (synovial sarcoma) | Wild-type | | |

### DNA probes

Hybridization probes were isolated from plasmids containing: 0.6-kb *Pst*I cDNA fragment from human retinoic acid recepter (RAR)-α (clone p63) in pTZ19R (ATCC); 0.6-kb *Eco*RI cDNA fragment from human RAR-β in pSG5 (kind gift from Dr P Chambon, INSERM, Illkirch, France); 1.3-kb *Eco*RI/*Ava*I cDNA fragment from human RAR-γ in pSG5 (kind gift from Dr P Chambon); 1.3-kb *Eco*RI/*Hin*dIII cDNA fragment from rat glyceraldehyde 3-phosphate dehydrogenase (GAPDH) in pSP65. Isolation of inserts were performed as described previously (Harant et al, 1995).

### Cell proliferation assay [($^3$H) Thymidine incorporation assay]

Tumour cells were plated in 96-well microtitre plates (Costar, Cambridge, MA, USA) at a density of $5 \times 10^4$ cells per well. Subsequently, retinoid and/or IFN-α-2b preparations in varying concentrations were added to tumour cell lines (see above), which had adhered for 1 h, and subsequently cultured for 48, 72, 96 and 120 h at 37°C under the appropriate conditions (HTB-82 cells were cultured in a humidified atmosphere containing 5% $CO_2$, whereas all other cell lines were cultured in a humidified

**Table 2** Correlation of p53-status with proliferation inhibition and apoptosis

| Cell line | p53 Mutation[a] | Proliferation inhibition | | Apoptosis induction | | |
|---|---|---|---|---|---|---|
| | | Interferon-α | Retinoids | 9cRA | tRA | 13cRA |
| HTB-82 (rhabdomyosarcoma) | No | Yes | Yes | No | Yes | No |
| HTB-91 (fibrosarcoma) | Yes | Yes | No | No | No | No |
| HTB-92 (liposarcoma) | Yes | Yes | No | No | No | No |
| HTB-93 (synovial sarcoma) | No | Yes | Yes | Yes | Yes | No |
| HTB-94 (chondrosarcoma) | Yes | Yes | No | No | No | No |

[a]As assessed by p53 sequence analysis.

© 1999 Cancer Research Campaign 

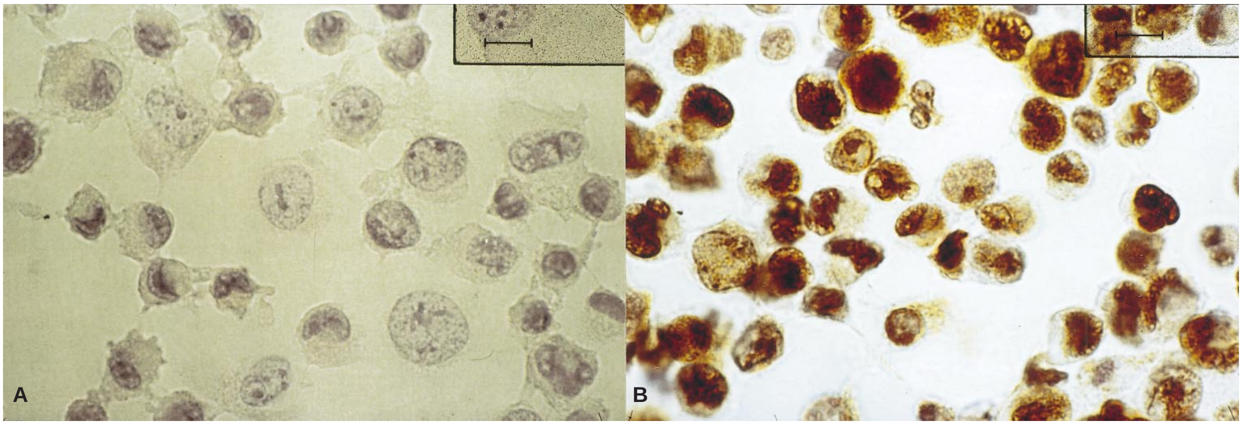

**Figure 1** Immunohistochemistry of soft tissue sarcoma cell lines for p53 protein. No overexpression of p53 was found in cell line HTB-91 (fibrosarcoma: **A**); whereas cell line HTB-92 (liposarcoma) showed intense nuclear staining (**B**)

atmosphere without $CO_2$). Medium containing retinoids and/or IFN-α-2b was replaced every 24 h. ($^3$H)Thymidine (Amersham International Life Science, plc, Buckinghamshire, UK), at a concentration of 0.5 μCi per well, was included for the final 16 h. The incorporation of ($^3$H)Thymidine into DNA was measured by a Direct Beta Counter-Matrix 96 (Packard, Groningen, The Netherlands) after the cells were harvested with the Harvester Micromate 196 (Packard, Groningen, The Netherlands) onto Glass Fiber Filters (Packard, Groningen, the Netherlands). Experiments were always done in triplicate. Data are presented as a percentage of proliferation of untreated cells for each respective time point.

### DNA fragmentation assay analysed by flow cytometry

The 3´OH termini in DNA breaks were measured by attaching fluorescent tagged deoxyuridine triphosphate nucleotides FITC-dUTP, in a reaction catalysed by terminal deoxynucleotidyl transferase (TdT) using the Apo-Direct™ Kit (Phoenix Flow Systems, San Diego, CA, USA) purchased from Pharmingen (San Diego, CA, USA). The amount of incorporated fluorescein was detected by flow cytometry.

HTB-82, HTB-91, HTB-92, HTB-93 and HTB-94 soft tissue sarcoma cell lines ($1 \times 10^6$ in T-25 flasks) were incubated with 9cRA, 13cRA or tRA (final concentrations: $10^{-7}$ M, $10^{-6}$ M, $10^{-5}$ M) for 48, 72, 96 and 120 h respectively. In addition, the same experiments were performed with IFN-α-2b (final concentration: 1000 U ml$^{-1}$) ± 9cRA, 13cRA or tRA (final concentrations: $10^{-7}$ M, $10^{-6}$ M, $10^{-5}$ M).

Untreated and treated cells were harvested, washed twice in phosphate-buffered saline (PBS), fixed in 1% (w/v) paraformaldehyde in PBS (pH 7.2), for 15 min on ice. After sedimentation and two more washing steps, cells were resuspended in ice-cold 70% (v/v) ethanol and stored at –20°C until further use (maximum: 3 weeks). According to the manufacturer's instructions, cells were washed twice in wash buffer, resuspended in 50 μl staining solution (10 μl reaction buffer, 0.75 μl TdT, 8 μl FITC-dUTP and 32 μl distilled water) and incubated for 1 h at 37°C. Afterwards, 1 ml rinsing buffer was added. Cells were centrifuged (1000 ***g***) and rinsed again. Subsequently, cells were resuspended in 1 ml propidium iodine (PI)/RNAase solution and incubated in the dark for 30 min at room temperature. Subsequently, cell samples were analysed by flow cytometry on a FACScan (Becton Dickinson, CA, USA).

### Morphological evaluation of apoptosis

Cell morphology was performed by staining with May–Gruenwald–Giemsa. For this reason, cells were cultured in chamber slides ('Lab-Tek Chamber Slide w/cover Glass Slide 2 Well') (Nalge Nunc International, Naperville, USA). Treated and untreated cells were stained with May–Gruenwald (Merck, Darmstadt, Germany) for 5 min. Subsequently, cells were washed with aqua dest (Leopold Pharma, Graz, Austria) for 5 min and stained with Giemsa (Merck, Darmstadt, Germany) (diluted 1:10) for 20 min. After a final wash with aqua dest, samples were viewed under light microscopy. Cells were considered to be apoptotic according to the criteria introduced by Kerr et al (1972) which included: compaction of the nuclear chromatin, fragmentation of nuclei, condensation of the cytoplasm and separation of the cell into apoptotic bodies.

### p53 immunohistochemistry

Immunohistochemical staining for the p53 protein was carried out using a mouse monoclonal IgG2a antibody (Clone: DO-1; Immunotech, Marseille, France; diluted 1:20) directed against wild-type and mutant p53 protein. For p53 staining, cytocentrifugates were fixed with Merckofix fixation spray (Merck, Darmstadt, Germany). After blocking with horse serum, samples were incubated with the primary antibody for 1 h. Further immunohistochemical staining was performed according to the ABC-method, using products from Vector Laboratories (Burlingame, CA, USA). Briefly, after incubation with the primary antibody and incubation with a biotinylated antimouse antibody, incubation with the ABC complex for 45 min followed. The reaction product was developed with diaminobenzidine tetrahydrochloride. Finally, slides were counterstained with Gill's haematoxylin. All steps of incubation were performed at room temperature.

### p53 sequence analysis

Total genomic tumour DNA was extracted from $2 \times 10^7$ cells using

 *© 1999 Cancer Research Campaign*

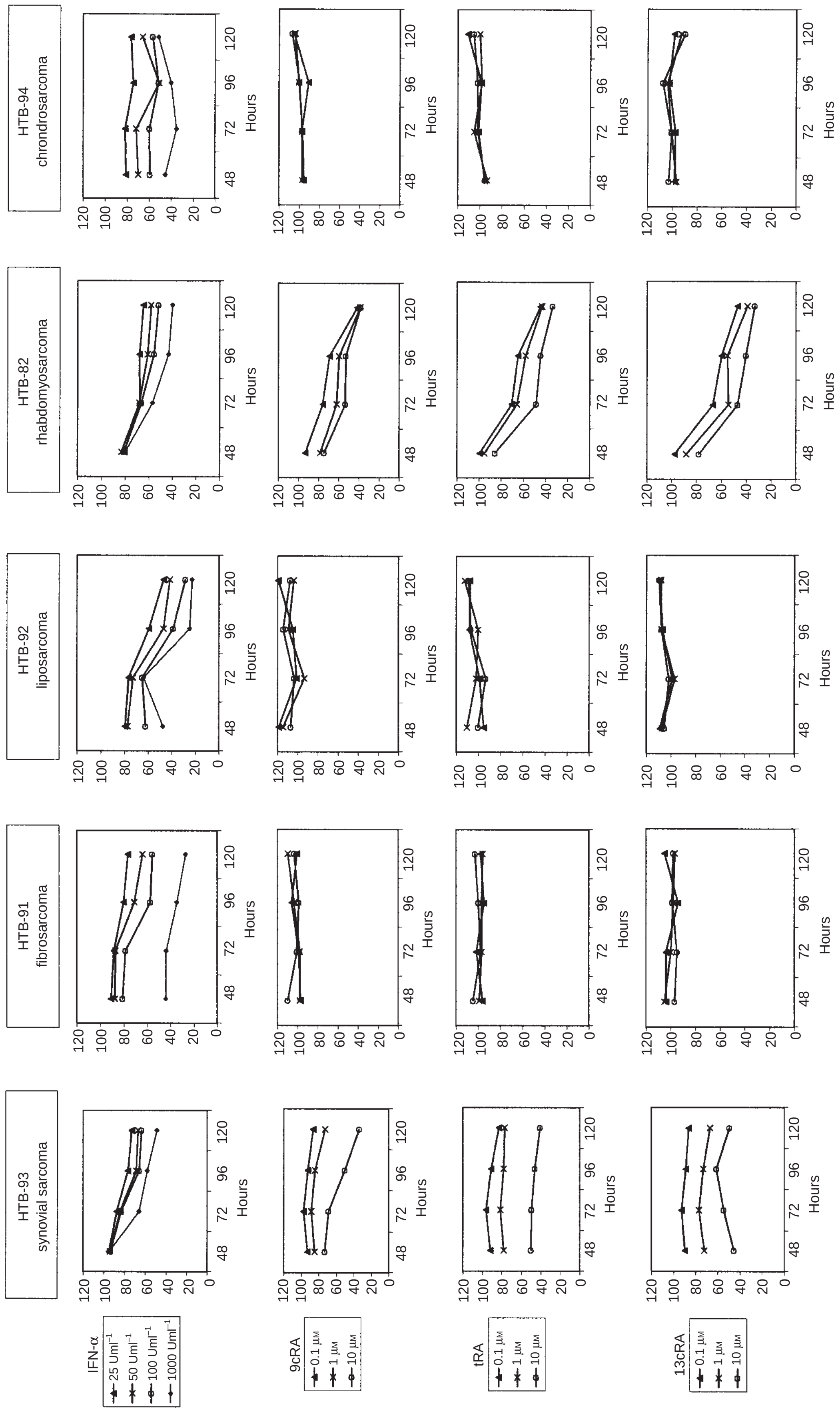

**Figure 2** Proliferation of soft tissue sarcoma cell lines during incubation with varying concentrations of the following substances ($^3$H-thymidine incorporation assay): lane 1: interferon-α; lane 2: 9-*cis*-retinoic acid; lane 3: all-*trans*-retinoic acid; lane 4: 13-*cis*-retinoic acid. Data are presented as relative counts per minute of untreated cells (100%) for each respective time point

*© 1999 Cancer Research Campaign*

standard phenol–chloroform extraction methods. Exons 2–11 of the p53 gene were amplified separately using oligonucleotide primers placed in the adjacent intron regions as described previously (Lehman et al, 1991). Polymerase chain reaction (PCR) products were controlled for purity, quantity and quality by subjecting 5 µ1 of PCR products to pre-cast 6% acrylamide/*bis*-acrylamide gels (Novex, San Diego, CA, USA) using the pBR322 DNA-*Msp*I digest as reference standard (Clontech Labs Inc., Palo Alto, CA, USA). To remove residual single-stranded primers, 5 µl of PCR products were enzymatically treated with combination of exonuclease I and shrimp alkaline phosphatase (United States Biochemical, Cleveland, OH, USA). Pretreated PCR products were then directly sequenced using the Cycle Sequencing Kit (Roche Molecular Systems, Inc. Branchburg, NJ, USA), utilizing Ampli *Taq*$^R$ DNA Polymerase and $\alpha^{35}S$ labelled dATP (DuPont NEN, Brussel, Belgium). For each reaction (for the four nucleotides), 0.5 µl of the thermostable DNA polymerase provided in the kit were added at last to the reaction mix containing 2 µl reaction buffer, 2–4 µl of enzymatically pretreated PCR product, 0.5 pmol unique primer (separate reaction for each exon) and water to adjust total volume to 20 µl. After running, the 8% acrylamide/*bis*-acrylamide gels are dried for 90 min at 70°C and directly subjected to autoradiography against the Bio Max-MR film (Kodak, New Haven, CT, USA). Mutations found were confirmed by at least one complete reanalysis.

### Detection of bcl-2 by immunofluorescence

Cells ($1 \times 10^6$ in T-25 flasks) were incubated with 9cRA, 13cRA or tRA (final concentrations: $10^{-7}$ M, $10^{-6}$ M, $10^{-5}$ M) for 48, 72, 96 and 120 h respectively. In addition, the same experiments were performed with IFN-α-2b (final concentration: 1000 U ml$^{-1}$) ± 9cRA, 13cRA or tRA (final concentrations: $10^{-7}$ M, $10^{-6}$ M, $10^{-5}$ M). After harvesting and three washes with PBS cells were fixed and permeabilized with the fix and perm cell permeabilization kit (An der Grub, Bio Research Gmbh, Kaumberg, Austria). According to the manufacturer's instructions, $10^6$ cells per sample were resuspended in 100 µl fixation medium and incubated for 15 min at room temperature. Afterwards, 5 ml PBS were added. Cells were centrifuged (5 min at 300 ***g***) and subsequently resuspended in 100 µl permeabilization medium and 10 µl FITC-conjugated monoclonal mouse $IgG_1$ anti-human bcl-2 antibody (clone 124; DAKO, Glostrup, Denmark), vortexed at low speed for 2 s and incubated for 15 min at room temperature. After one more washing step, cells were analysed by flow cytometry on a FACScan (Becton Dickinson, CA, USA).

### Northern blotting for RAR mRNA expression

STS cells were plated in T-75 tissue culture flask (Costar) and were grown to sub-confluence (see above). The total RNA was extracted by a one-step acid guanidinium isothiocyanate–phenol procedure using RNAzol (Biotecx, Houston, TX, USA) and 20 mg of the RNA were loaded into each lane and were electrophoresed for 4 h at 50 V in a 1% formaldehyde-containing agarose gel. The separated RNA was then diffusion-blotted overnight onto nylon membranes (Schleicher & Schuell, Dassel, Germany) and immobilized by UV-cross-linking. RAR- and GAPDH-specific cDNA probes were labelled with $^{32}P$-dCTP (New England Nuclear, Vienna, Austria) using the random oligonucleotide primer extension kit (Stratagene, La Jolla, CA, USA). The membranes were hybridized overnight at 65°C, washed twice with 2 × saline-sodium citrate (SSC) and 0.1% sodium dodecyl sulphate (SDS) at 37°C and twice with 0.1 × SSC and 0.1% SDS at 65°C and were exposed at –80°C to Kodak X-OMAT AR film (Eastman Kodak, Rochester, NY, USA) between two intensifying screens. The blots were then stripped and reprobed (overnight, 65°C) with the GAPDH cDNA as an internal control to monitor the quantity and integrity of the loaded RNA samples.

### RESULTS

### p53 genotype and protein expression

Native cell lines HTB-82, HTB-91, HTB-92, HTB-93 and HTB-94 were screened for their p53 genotype as well as their p53 protein expression. p53 gene mutations are characterized in Table 1: HTB-91, HTB-92 and HTB-94 cell lines had mutations in the p53 gene, whereas wild-type p53 was found in HTB-82 and HTB-93 cell lines. Immunohistochemistry for p53 revealed intense nuclear staining in HTB-92 (Figure 1B) and HTB-94 cells, whereas HTB-82, HTB-91 (Figure 1A) and HTB-93 cells showed no nuclear staining. The p53 mutation of HTB-91 cells was probably associated with loss of protein staining.

### Proliferation inhibition

To test the influence of retinoids and IFN-α on proliferation, STS cell lines were incubated with IFN-α and/or retinoids for 48, 72, 96 and 120 h respectively. Figure 2 shows that IFN-α dose- and time-dependently inhibited the proliferation of all five STS cell lines at concentrations ranging from 25 U ml$^{-1}$ to 1000 U ml$^{-1}$ and incubation periods from 48 to 120 h. It has to be noted, however, that the inhibitory potential of IFN-α exerted upon various tested cell lines varied with ranges between 49 and 77% (Figure 2).

Similarly, 9cRA, 13cRA or tRA showed a dose- ($10^{-7}$ M, $10^{-6}$ M and $10^{-5}$ M) and time-dependent proliferation inhibitory effect on cell lines HTB-82 and HTB-93 (Figure 2). In contrast, HTB-91, HTB-92 and HTB-94 could not be inhibited in their proliferation by either 9cRA or 13cRA or tRA (Figure 2). Coincubation of HTB-82, HTB-91, HTB-92, HTB-93 and HTB-94 STS cells with retinoids and IFN-α did not further increase the antiproliferative effect of IFN-α (data not shown). In control experiments, DMSO only had no inhibitory influence upon either of the five cell lines after any duration of incubation.

### Induction of apoptosis: DNA fragmentation, analysis of cell cycle position and DNA content

Induction of apoptosis by retinoids or IFN-α in STS cell lines was investigated by DNA strand-break analysis. In order to analyse cell cycle position of STS cells, global DNA content was measured with PI counterstaining. HTB-82, HTB-91, HTB-92, HTB-93 or HTB-94 STS cells were incubated with 9cRA, 13cRA or tRA (final concentrations: $10^{-7}$ M, $10^{-6}$ M and $10^{-5}$ M) and/or IFN-α (1000 U ml$^{-1}$) for 48, 72, 96 or 120 h respectively. DNA histograms obtained from STS cell lines and the respective percentages of apoptotic and non-apoptotic cells after 96 h incubation with $10^{-5}$ M of the appropriate retinoid are shown in Figure 3. In summary, apoptosis could be induced by retinoids 9cRA or tRA in cell lines HTB-82 and HTB-93 dose-dependently with an optimal duration of incubation with $10^{-5}$ M of the appropriate retinoid for 96 h (Figure 3), whereas no apoptosis was found in cell lines HTB-91, HTB-92 and HTB-94 during an

 © 1999 Cancer Research Campaign

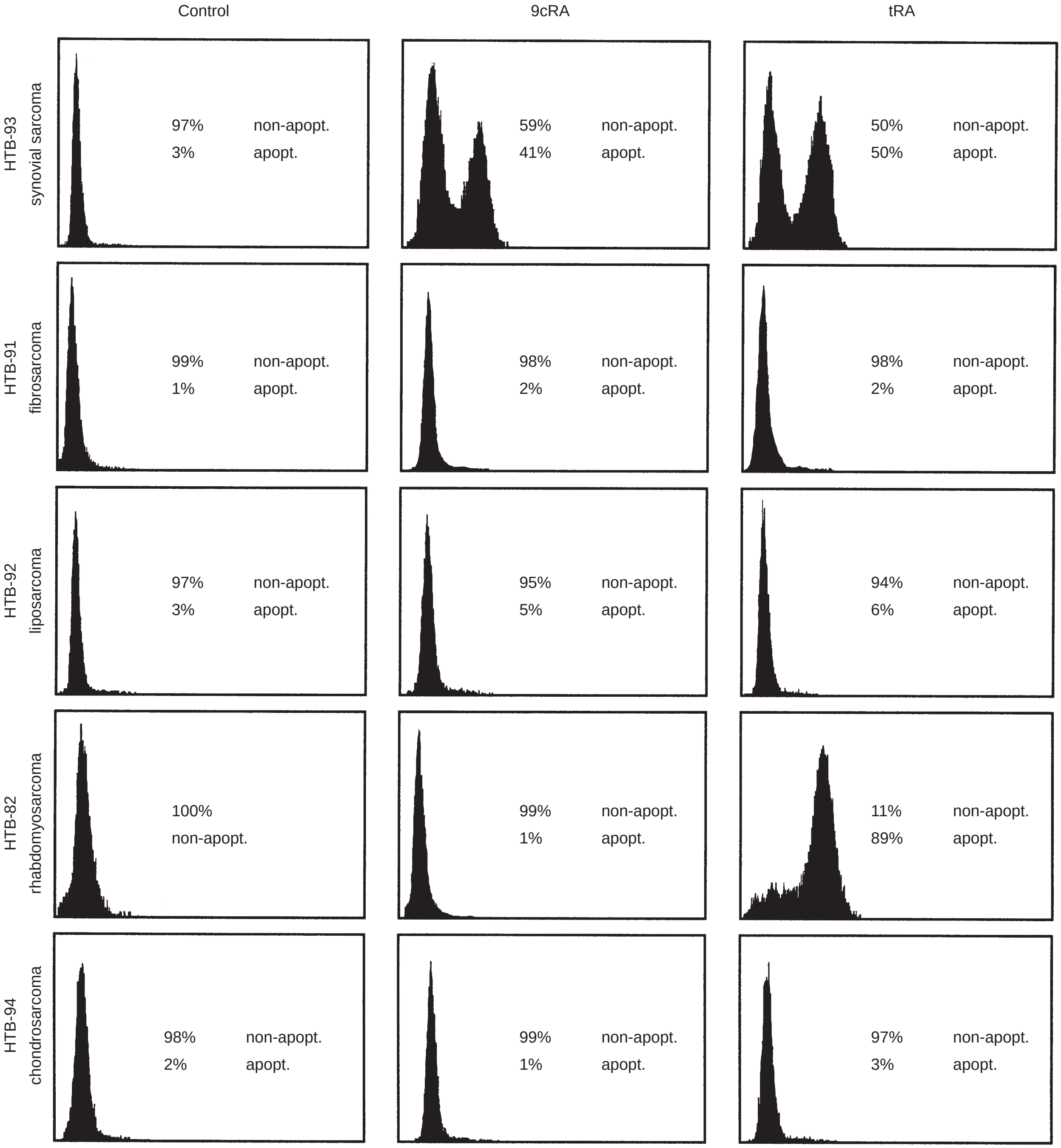

**Figure 3** FACS histograms of specifically for apoptosis stained soft tissue sarcoma cell lines after incubation without (control) or with $10^{-5}$ M 9-*cis*-retinoic acid (9cRA) or all-*trans*-retinoic acid (tRA) for 96 h. FACS histograms show on the *x*-axis the log fluorescence intensity of non-apoptotic and apoptotic cells within the tested cell population. The *y*-axis represents the relative number of cells incubation period of up to 120 h. In addition, treatment of HTB-82 and HTB-93 cells with 9cRA or tRA was associated with some G1–S arrest. Finally, neither 13cRA nor IFN-α induced apoptosis in either cell line. In detail, the results of induction of apoptosis in correlation with cell cycle positions were as follows.

### *HTB-93 (synovial sarcoma)*

After 96 h of incubation with 9cRA ($10^{-5}$ M), the percentage of apoptotic cells was 41% (G1: 35%; G2 and S: 6%) versus 59% non-apoptotic cells (G1: 43%; G2 and S: 16%). At lower doses of 9cRA ($10^{-6}$ M and $10^{-7}$ M) we observed 29 and 20% apoptotic cells respectively. After incubation with tRA ($10^{-5}$ M) 50% of the cells (G1: 45%; G2 and S: 5%) underwent apoptosis versus 50% non-apoptotic cells (G1: 38%; G2 and S: 12%). At a concentration of $10^{-6}$ M of tRA, 36% of cells showed apoptosis and 25% at $10^{-7}$ M. No apoptosis was induced by either retinoid by incubation for 48 h.

### *HTB-82 (rhabdomyosarcoma)*

Only tRA was capable of inducing apoptosis. After 96 h of incubation 89% (G1: 67%; G2 and S: 22%) of the cells were apoptotic.

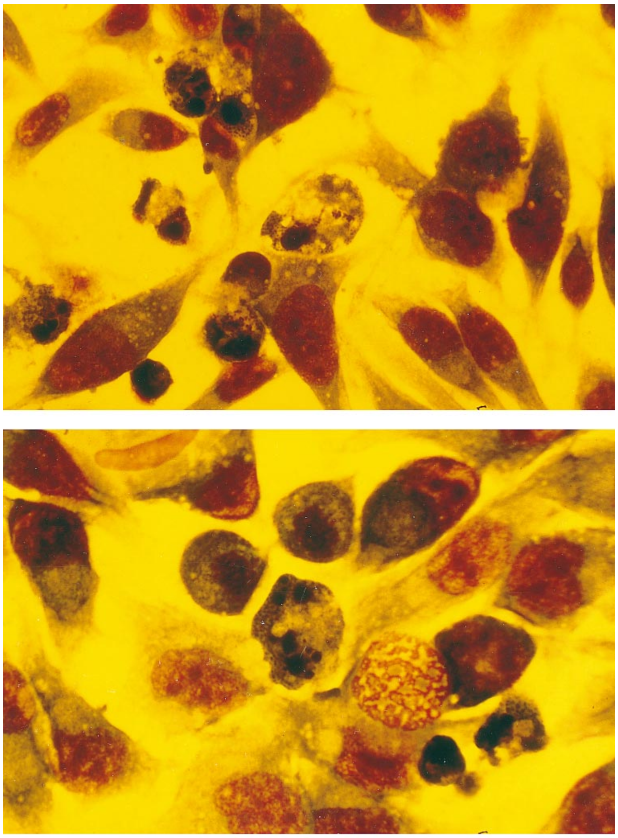

**Figure 4** May–Gruenwald–Giemsa staining of HTB-82 cells (rhabdomyosarcoma) after incubation with all-*trans*-retinoic acid ($10^{-5}$ M) for 72 h. Cells show typical apoptotic features such as nuclear-chromatin compaction, cytoplasma condensation around the nucleus, cell shrinkage and apoptotic ‘bodies’

Eleven per cent of the cells did not undergo apoptosis (G1: 9%; G2 and S: 2%). At lower concentrations of tRA ($10^{-6}$ M and $10^{-7}$ M) we observed 50 and 41% apoptotic cells respectively.

*HTB-91 (fibrosarcoma), HTB-92 (liposarcoma) and HTB-94 (chondrosarcoma)*

No apoptosis was measured after treatment with 9cRA, 13cRA, tRA and/or IFN-α. Neither 13cRA nor IFN-α in any concentration ($10^{-7}$–$10^{-5}$ M; 25–1000 U $ml^{-1}$) and after any length of incubation (48–120 h) were capable of inducing apoptosis in either cell line. Moreover, the addition of IFN-α to retinoids which were able to induce apoptosis did not increase their apoptotic potency (data not shown). Finally, incubation of all five cell lines with DMSO only did not induce apoptosis after any time interval (data not shown).

### Morphologic evaluation of apoptosis

Morphology of cells shown to undergo apoptosis by flow cytometry exhibited typical apoptotic features such as nuclear-chromatin compaction, cytoplasma condensation around the nucleus, cell shrinkage and apoptotic ‘bodies’ (Figure 4).

### bcl-2 protein expression

The effects of 9cRA, 13cRA and tRA on bcl-2 protein expression were also studied. All five native cell lines (HTB-82, HTB-91, HTB-92, HTB-93 and HTB-94) weakly expressed bcl-2 protein (10–20%). Treatment of these cell lines with 9cRA, 13cRA and tRA (final concentrations: $10^{-7}$ M, $10^{-6}$ M, $10^{-5}$ M) for 48, 72, 96 and 120 h respectively, did not modify the expression of bcl-2 protein.

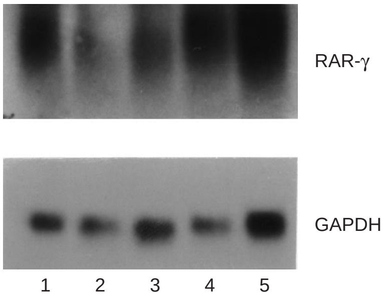

**Figure 5** RAR-γ mRNA expression in five different human soft tissue sarcoma cell lines. Northern blot analysis was performed on 20 mg RNA using a $^{32}$P-labelled RAR-γ cDNA probe (upper panel). After autoradiography, the membranes were stripped and reprobed with a $^{32}$P-labelled GAPDH cDNA as an internal loading control (lower panel). Lane 1: HTB-92; lane 2: HTB-93; lane 3: HTB-91; lane 4: HTB-94; lane 5: HTB-82

### Expression of RAR isoforms

None of the five STS cell lines expressed RAR-α and RAR-β mRNA at levels detectable by Northern blot analysis. However, RAR-γ-specific transcripts with molecular sizes of approximately 3.3 kb were found in variable amounts in all cell lines (Figure 5). Thus, the differential response of STS cell lines to retinoids was not associated with a lack of RARs in general, or with a defect in the expression of the universal retinoid receptor RAR-γ in particular, or with a specific pattern of RAR isoform expression.

### Correlation of p53 status with proliferation inhibition and apoptosis

Normal p53 was found in cell lines in which retinoids 9cRA or tRA were capable of inducing apoptosis and in which retinoids 9cRA, 13cRA and tRA led to proliferation inhibition. In contrast, mutant p53 was present in cell lines in which retinoids were not able to induce either apoptosis or proliferation inhibition (Table 2). However, proliferation inhibition induced by IFN-α occurred in all cell lines irrespective of their p53 status (Table 2). In control experiments, p53 genotype as well as p53 protein expression did not change after incubation of STS cell lines HTB-82,-91, -92, -93 and -94 with 9cRA, 13cRA or tRA (final concentrations: $10^{-7}$ M, $10^{-6}$ M and $10^{-5}$ M) and/or IFN-α (final concentrations: 25, 50, 100 and 1000 U $ml^{-1)}$ for 48, 72, 96 or 120 h (data not shown).

### DISCUSSION

In response to DNA damage, wild-type p53 has been shown to induce either cell cycle arrest in the G1 phase (Kastan et al, 1991; Di Leonardo et al, 1994), allowing for DNA repair, or apoptosis (Yonish-Rouach et al, 1991). In addition, wild-type p53 is also responsible for the regulation of the cell cycle (Harvey et al, 1993) during which p53-mediated arrests have been reported to occur not only in the G1, but also in the G2 phases (Ryan et al, 1993; Agarwal et al, 1995; Stewart et al, 1995). The final decision whether induction of p53 triggers G1 arrest or apoptosis

 *© 1999 Cancer Research Campaign*

(Slichenmyer et al, 1993) is thought to be dependent on the presence of a growth factor which provides a survival signal (Canman et al, 1995). However, additional p53-independent apoptotic pathways exist in normal as well as malignant cells which differ in their sensitivity towards apoptosis-inducing agents (Delia et al, 1993; Bracey et al, 1995; Shao et al, 1995; Thompson, 1995).

Retinoids exert a broad spectrum of biologic effects, including inhibition of cell growth and induction of apoptosis in a variety of cancer cell lines (Lotan, 1995). These biological effects are thought to result from modulation of gene expression mediated by nuclear receptors, including RARs which are expressed in three different isoforms (RAR-α, RAR-β and RAR-γ; Mangelsdorf et al, 1995). Although it has been generally accepted that retinoid-triggered apoptosis mainly involves p53-dependent pathways (Lovat et al, 1997; Toma et al, 1997), recent reports have provided evidence about the possibility of retinoid-induced apoptosis independent of p53 (Dmitrovsky, 1997; Giandomenico et al, 1997; Oridate et al, 1997).

In the present investigation, we have shown that the mechanisms of proliferation inhibition and induction of apoptosis in cells derived from STS lines varying in p53 mutations can act independently from the used agents. Thus, IFN-α was found to induce inhibition of proliferation independently from the cells' p53 status, whereas the inhibition of proliferation achieved by retinoids was associated with intact p53 and was not found in cells with p53 mutations. Considering its important regulatory potential upon blocking apoptosis (Hockenbery et al, 1990), no influence of bcl-2 within the context of the reported findings was detectable in the present model. Nevertheless, the degree of inhibition of proliferation varied between cell lines depending upon the chosen retinoid, its concentration and time. In order to illustrate the actual inhibition of cellular proliferation rather than the induction of apoptosis, a thymidine incorporation assay was used which proved DNA synthesis occurred thus providing a direct parameter for cellular proliferation and its actual inhibition. These findings suggest that p53 status and its influence upon proliferation inhibition in STS cell lines acted independently from and changed with the used preparation. It is conceivable, therefore, that proliferation inhibition induced by IFN-α used a different pathway independent from p53 (Dmitrovsky, 1997; Giandomenico et al, 1997; Oridate et al, 1997) the functionality of which is important, however, for the activity exerted by retinoids in the same context.

In contrast to its antiproliferative effect, IFN-α did not have the ability to induce apoptosis in the tested STS cell lines. In contrast, tRA was capable of inducing apoptosis in cell lines HTB-82 and HTB-93, and 9cRA in cell line HTB-93, whereas 13cRA did not induce apoptosis, at all. However, no induction of apoptosis by otherwise effective retinoids 9cRA or tRA occurred in cell lines HTB-91, HTB-92 and HTB-94 in which mutations of p53 were found. Data obtained from Northern blot analysis demonstrated that resistance to induction of apoptosis induced by retinoids in the three STS cell lines harbouring mutations of the p53 gene did not result from a defect in the expression of RARs indicating that p53 did not regulate RAR-α, -β or -γ expression in these instances. As RAR-γ constitutes a universal receptor for the used retinoids (Repa et al, 1997; Fisher et al, 1998), a defect in the presence of a relevant receptor can be excluded as an explanation for our findings. Thus, no simple correlation of retinoid sensitivity with the expression pattern of RARs could be established for STS cell lines which is in accordance with previous reports (Van der Leede et al, 1993; Engers et al, 1996). Although p53-mutated HTB-92 and HTB-94 cell lines showed intensive nuclear staining by immunohistochemistry, the p53 mutated HTB-91 cell line showed no nuclear staining. The p53 mutation in HTB-91 is probably related to a loss of protein staining (Aas et al, 1996). It is, therefore, interesting to note that the inhibition of proliferation by retinoids was closely linked to their ability to induce apoptosis, suggesting the importance of p53 in both contexts. It is noteworthy, however, that 13cRA – while being able to inhibit cellular proliferation – did not induce apoptosis, thus further suggesting a possible divergence of mechanisms regulating the two phenomena. This assumption is supported by recent reports that have demonstrated that 13cRA did not bind to RARs on its own (Wu et al, 1994; Berggren et al, 1995). In summary, an overview of our data suggests that only mutations of p53 correlated with an abrogation of proliferation inhibition and of induction of apoptosis by retinoids 9cRA and tRA.

Regarding the possible clinical impact of our data, we report an antiproliferative influence of IFN-α and certain retinoids as well as the induction of apoptosis by the latter group of drugs, which could constitute the basis for clinical studies in patients with STS, especially when cytotoxic chemotherapy has failed. In similarity to this assumption, recent reports have already reported about the successful inclusion of retinoids in the treatment of certain malignancies (Tallman et al, 1997). Concerning STS, however, our results have shown that the histological subtype and p53 status might predict the efficacy of this therapy. The latter aspect could also be valid for the use of cytotoxic therapy which often has failed to produce convincing results in STS in the adjuvant or palliative setting.

### ACKNOWLEDGEMENTS

The authors gratefully acknowledge expert technical assistance by Ms Waclawa Kalinowski.

This study was supported in part by a donation in memory of the late Mr. Alfred Matejka and by a grant from the 'Medizinisch-Wissenschaftlicher Fonds des Bürgermeisters der Bundeshauptstadt Wien'.

### REFERENCES

Aas T, Borresen AL, Geisler S, Smith-Sorensen B, Johnson H, Varhaug JE, Akslen LA and Lonning PE (1996) Specific p53 mutations are associated with de novo resistance to doxorubicin in breast cancer patients. *Nat Med* **2**: 811–814

Agarwal ML, Agarwal A, Taylor WR and Stark GR (1995) p53 controls both the G2/M and the G1 cell cycle checkpoints and mediates reversible growth arrest in human fibroblasts. *Proc Natl Acad Sci USA* **92**: 8493–8497

Antman K, Crowley J, Balcerzak SP, Rivkin SE, Weiss GR, Elias A, Natale RB, Cooper RM, Barlogie B, Trump DL, Doroshow JH, Aisner J, Pugh RP, Weiss RB, Cooper BA, Clamond GH and Baker LH (1993) An intergroup phase III randomized study of doxorubicin and dacarbacine with or without ifosfamide and mesna in advanced soft tissue and bone sarcomas. *J Clin Oncol* **11**: 1276–1285

Antman KH (1997) Adjuvant therapy of sarcomas of soft tissue. *Semin Oncol* **24**: 556–560

Berggren Soderlund M, Johannesson G and Fex G (1995) Expression of human all-trans-retinoic acid receptor beta and its ligand-binding domain in *Escherichia coli*. *Biochem J* **308**: 353–359

Bergh J, Norberg T, Sjogren S, Lindgren A and Holmberg L (1995) Complete sequencing of the p53 gene provides prognostic information in breast cancer patients, particularly in relation to adjuvant systemic therapy and radiotherapy. *Nat Med* **1**: 1029–1034

Borden EC, Amato DA, Edmonson JH, Ritch PS and Shiraki M (1990) Randomized comparison of doxorubicin and vindesine to doxorubicin for patients with metastatic soft-tissue sarcomas. *Cancer* **66**: 862–867

Bracey TS, Miller JC, Preece A and Paraskeva C (1995) γ-Radiation-induced apoptosis in human colorectal adenoma and carcinoma cell lines can occur in the absence of wild-type p53. *Oncogene* **10**: 2391–2396
Brodowicz T, Kotz R, Wiltschke C, Zielinski CC and Ritschl P (1997) Re: isolated limb perfusion with high-dose tumor necrosis factor-alpha in combination with interferon-γ and melphalan for non-resectable extremity soft tissue sarcomas: a multicenter trial. *J Clin Oncol* **15**: 2174–2175
Canman CE, Gilmer TM, Coutts SB and Kastan MB (1995) Growth factor modulation of p53-mediated growth arrest versus apoptosis. *Gene Dev* **9**: 600–611
Crouch GD and Helman LJ (1991) All-*trans*-retinoic acid inhibits the growth of human rhabdomyosarcoma cell lines. *Cancer Res* **51**: 4882–4887
Delia D, Aiello A, Lombardi L, Pelicci PG, Grignani Fr, Grignani Fa, Formelli F, Menard S, Costa A, Veronesi U and Pierotti MA (1993) N-(4-hydroxyphenyl)retinamide induces apoptosis of malignant hemopoietic cell lines including those unresponsive to retinoic acid. *Cancer Res* **53**: 6036–6041
Di Leonardo A, Linke SP, Clarkin K and Wahl GM (1994) DNA damage triggers a prolonged p53-dependent G1 arrest and long-term induction of *Cip*1 in normal human fibroblasts. *Genes Dev* **8**: 2540–2551
Dmitrovsky E (1997) *N*-(4-hydroxyphenyl)retinamide activation of a distinct pathway signaling apoptosis. *J Natl Cancer Inst* **89**: 1179–1181
Elledge RM, Gray R, Mansour E, Yu Y, Clark GM and Ravdin P (1995) Accumulation of p53 protein as a possible predictor of response to adjuvant combination chemotherapy with cyclophosphamide, methotrexate, fluorouracil, and prednisone for breast cancer. *J Natl Cancer Inst* **87**: 1254–1256
Engers R, van Roy F, Heymer T, Ramp U, Moll R, Dienst M, Friebe U, Pohl A, Gabbert HE and Gerharz CD (1996) Growth inhibition in clonal subpopulations of a human epithelioid sarcoma cell line by retinoic acid and tumour necrosis factor alpha. *Br J Cancer* **73**: 491–498
Fisher GJ, Datta SC and Voorhees JJ (1998) Retinoic acid receptor-γ in human epidermis preferentially traps all-*trans* retinoic acid as its ligand rather than 9-*cis* retinoic acid. *J Invest Dermatol* **110**: 297–300
Gabbert HE, Gerharz CD, Biesalski HK, Engers R and Luley C (1988) Terminal differentiation and growth inhibition of a rat rhabdomyosarcoma cell line (BA-HAN-1C) in vitro after exposure to retinoic acid. *Cancer Res* **48**: 5264–5269
Gherlinzoni F, Bacci F, Picci P, Capanna R, Calderoni P, Lorenzi EG, Bernini M, Emiliani E, Barbieri E, Normand A and Campanacci M (1986) A randomized trial for the treatment of high-grade soft-tissue sarcomas of the extremities: preliminary observations. *J Clin Oncol* **4**: 552–558
Giandomenico V, Lancillotti F, Fiorucci G, Percario ZA, Rivabene R, Malorni W, Affabris E and Romeo G (1997) Retinoic acid and IFN inhibition of cell proliferation is associated with apoptosis in squamous carcinoma cell lines: role of IRF-1 and TGase II-dependent pathways. *Cell Growth Differ* **8**: 91–100
Harant H, Lindley I, Uthman A, Ballaun C, Kruptiza G, Grunt T, Huber H and Dittrich C (1995) Regulation of interleukin-8 gene expression by all-*trans* retinoic acid. *Biochem Biophys Res Commun* **210**: 898–906
Harvey M, Sands AT, Weiss RS, Hegi ME, Wiseman RW, Pantazis P, Giovanella BC, Tainsky MA, Bradley A and Donehower LA (1993) In vitro growth characteristics of embryo fibroblasts isolated from p53-deficient mice. *Oncogene* **8**: 2457–2467
Hockenbery D, Nunez G, Milliman C, Schreiber RD and Korsmeyer SJ (1990) Bcl-2 is an inner mitochondrial membrane protein that blocks programmed cell death. *Nature* **348**: 334–336
Kastan MB, Onyekwere O, Sidransky D, Vogelstein B and Craig RW (1991) Participation of p53 in the cellular response to DNA damage. *Cancer Res* **51**: 6304–6311
Kerr JFR, Wyllie AH and Currie AR (1972) Apoptosis: a basic biological phenomenon with wide ranging implications in tissue kinetics. *Br J Cancer* **26**: 239–257
Lehman TA, Bennett WP, Metcalf RA, Welsh JA, Ecker J, Modali RV, Ullrich S, Romano JW, Appella E, Testa JR, Gerwin BI and Harris CC (1991) P53 mutations, ras mutations, and p53-heat shock 70 protein complexes in human lung carcinoma cell lines. *Cancer Res* **51**: 4090–4096
Lotan R (1995) Retinoids and apoptosis: implications for cancer chemoprevention and therapy. *J Natl Cancer Inst* **87**: 1655–1657
Lovat PE, Irving H, Annicchiarico-Petruzzelli M, Bernassola F, Malcolm AJ, Pearson AD, Melino G and Redfern CP (1997) Apoptosis of N-type neuroblastoma cells after differentiation with 9-*cis*-retinoic acid and subsequent washout. *J Natl Cancer Inst* **89**: 446–452
Lutzker SG and Levine AJ (1996) A functionally inactive p53 protein in teratocarcinoma cells is activated by either DNA damage or cellular differentiation. *Nature Med* **2**: 804–810
Mangelsdorf DJ, Thummel C, Beato M, Herrlich P, Schutz G, Umesono K, Blumberg B, Kastner P, Mark M, Chambon P and Evans RM (1995) The nuclear receptor superfamily: the second decade. *Cell* **83**: 835–839
Oridate N, Suzuki S, Higuchi M, Mitchell MF, Hong WK and Lotan R (1997) Involvement of reactive oxygen species in *N*-(4-hydroxyphenyl)retinamide-induced apoptosis in cervical carcinoma cells. *J Natl Cancer Inst* **89**: 1191–1198
Ravaud A, Nguyen BB and Coindre JM (1990) Adjuvant chemotherapy with CYVADIC in high-risk soft tissue sarcoma: a randomized prospective trial. In: *Adjuvant Therapy of Cancer VI*, Salmon SE (ed), pp. 556–566. WB Saunders: Philadelphia
Repa JJ, Berg JA, Kaiser ME, Hanson KK, Strugnell SA and ClagettDame M (1997) One-step immunoaffinity purification of recombinant human retinoic acid receptor γ. *Protein Expres Purif* **9**: 319–330
Rosenberg SA, Tepper J, Glatstein E, Costa J, Baker A, Brennan M, De Moss EV, Seipp C, Sindelar WF, Sugarbaker P and Wesley R (1982) The treatment of soft-tissue sarcoma of the extremities: prospective randomized evaluations of (1) limb-sparing surgery plus radiation therapy compared with amputation and (2) the role of adjuvant chemotherapy. *Ann Surg* **196**: 305–315
Rosso R, Sertoli MR, Queirolo P, Sanguineti O, Barzacchi MC, Mariani GL, Miglio L, Venturini M and Toma S (1992) An outpatient phase I study of a subcutaneous interleukin-2 and intramuscular α-2a-interferon combination in advanced malignancies. *Ann Oncol* **3**: 559–563
Ryan JJ, Danish R, Gottlieb CA and Clarke MF (1993) Cell cycle analysis of p53-induced cell death in murine erythroleukemia cells. *Mol Cell Biol* **13**: 711–719
Sarcoma Meta-analysis Collaboration (1997) Adjuvant chemotherapy for localised resectable soft-tissue sarcoma of adults: meta-analysis of individual data. *Lancet* **350**: 1647–1654
Sarkis AS, Bajorin DF, Reuter VE, Herr HW, Netto G, Zhang ZF, Schultz PK, Cordon-Cardo C and Scher HI (1995) Prognostic value of p53 nuclear overexpression in patients with invasive bladder cancer treated with neoadjuvant MVAV. *J Clin Oncol* **13**: 1384–1390
Shao ZM, Dawson MI, Li XS, Rishi AK, Sheikh MS, Han QX, Ordonez JV, Shroot B and Fontana JA (1995) p53 independent $G_0/G_1$ arrest and apoptosis induced by a novel retinoid in human breast cancer cells. *Oncogene* **11**: 493–504
Slichenmyer WJ, Nelson WG, Slebos RJ and Kastan MB (1993) Loss of a p53-associated G1 checkpoint does not decrease cell survival following DNA damage. *Cancer Res* **53**: 4164–4168
Stewart N, Hicks GG, Paraskevas F and Mowat M (1995) Evidence for a second cell cycle block at G2/M by p53. *Oncogene* **10**: 109–115
Tallman MS, Andersen JW, Schiffer CA, Appelbaum FR, Feusner JH, Ogden A, Shepherd L, Willman C, Bloomfield CD, Rowe JM and Wiernik PH (1997) All-*trans*-retinoic acid in acute promyelocytic leukemia. *N Engl J Med* **337**: 1021–1028
Thompson CB (1995) Apoptosis in the pathogenesis and treatment of disease. *Science* **267**: 1456–1462
Toma S, Isnardi L, Raffo P, Dastoli G, De Francisci E, Riccardi L, Palumbo R and Bollag W (1997) Effects of all-*trans*-retinoic acid and 13-*cis*-retinoic acid on breast-cancer cell lines: growth inhibition and apoptosis induction. *Int J Cancer* **70**: 619–627
van der Leede BM, van den Brink CE and van der Saag PT (1993) Retinoic acid receptor and retinoid X receptor expression in retinoic acid-resistant human tumor cell lines. *Mol Carcinog* **8**: 112–122
Wu YN, Gadina M, Tao-Cheng JH and Youle RJ (1994) Retinoic acid disrupts the

 *© 1999 Cancer Research Campaign*
